# Two-Layer Electroosmotic Flow in a Parallel Plate Microchannel with Sinusoidal Corrugation

**DOI:** 10.3390/mi15111315

**Published:** 2024-10-29

**Authors:** Long Chang, Mandula Buren, Geming Bai, Yanjun Sun, Yongjun Jian

**Affiliations:** 1 School of Statistics and Mathematics, Inner Mongolia University of Finance and Economics, Hohhot 010071, China; suolunga@163.com (L.C.); baigeming0@163.com (G.B.); sunyanjun.2006@163.com (Y.S.); 2 School of Mathematical Science, Inner Mongolia University, Hohhot 010021, China; 3 School of Mathematical Science, Inner Mongolia Normal University, Hohhot 010022, China; brmdllyc@163.com; 4 School of Mathematics and Statistics, Donghua University, Shanghai 201620, China; 5 Institute for Nonlinear Science, Donghua University, Shanghai 201620, China

**Keywords:** electroosmotic flow, electric double layer, sinusoidal corrugated wall, two-fluid pump, conducting fluid and nonconducting fluid

## Abstract

This study investigates the electroosmotic flow (EOF) of a two-layer Newtonian fluid system in a parallel plate microchannel with sinusoidal corrugated walls. The upper fluid is conducting, while the lower fluid is nonconducting. This analysis is performed under the Debye–Hückel approximation, utilizing perturbation expansion and the separation of variables. The potential distribution, velocity field, and the dependence of average velocity on roughness are derived. It is observed that the velocity distribution *w*(*x*, *y*), is significantly influenced by the phase difference *θ* between the corrugations on the upper and lower walls. The velocity *w*(*x*, *y*) decreases with an increase in the viscosity ratio *μ_r_* of the bottom to top fluid, and *w*(*x*, *y*) is directly proportional to the dimensionless pressure gradient *G* and the zeta potential ratio *ζ*. The variation of the average velocity increment (roughness function) *u*_2m_ related to wall roughness tends to decrease with the increase of the corrugation wave number *λ*, the electrokinetic width *K*, the depth ratio *h_r_* of the bottom to top fluid, the zeta potential ratio *ζ* and the dimensionless pressure gradient *G*; and increases with the increase of the viscosity ratio *μ_r_* of the bottom to top fluid. Furthermore, the effect of *u^I^*_2*m*_ is smaller than that of *u^II^*_2*m*_.

## 1. Introduction

The advancement of microfluidic technology has led to the widespread utilization of electroosmotic flow (EOF) in various applications within microfluidic chips, including DNA separation, cell sorting, ion transport, and sample separation and mixing [1]. EOF facilitates fluid movement within micro or nanoscale channels, boasting advantages such as low energy consumption, ease of operation, and the absence of external mechanical forces. Compared to flow in conventional-scale channels, microscale channels exhibit unique characteristics, including relative slip effects, surface roughness, surface forces, capillary effects, microscale effects, and rapid heat conduction [2]. Many researchers have conducted theoretical, numerical simulation, and experimental studies on EOF in Newtonian [3,4,5,6,7] and non-Newtonian fluids [8,9,10,11] within smooth microchannels of various geometries.

While monolayer EOF has been extensively researched and applied in various fields such as biotechnology, microfluidics, electrophoretic separation, and micro/nanofabrication, there are situations where precise control of particle motion is crucial, thus necessitating a focus on two-layer fluid flows. In monolayer fluids, particles are subject to fluid traction and flow constraints. However, in bilayer fluids, particles experience the combined influences of electric fields, chemical gradients, and hydrodynamics, resulting in more complex behavior than monolayer EOF. Due to the differing properties of the fluids, one layer may exert additional external forces (such as electroosmotic (EO) force or viscous shear force) on the particles. Therefore, current research on EOF offers deeper insights into particle motion behavior and presents more opportunities for precise manipulation and localization. Furthermore, two-layer EOF has practical applications; for instance, in microfluidic biochips, differences between the two layers of liquid can be leveraged for cell separation and classification.

Recent advances in microfluidic technology, including the development of microelectrical mechanical systems (MEMS), have enabled precise microscale operations such as the delivery, mixing, and separation of multiple liquid types. Despite these advancements, challenges remain with nonconductive fluids [12] like oil, blood, and ethanol, which have low electrical conductivity (<10^−6^ S m^−1^) and are not effectively moved by EO forces. Additionally, applying electric fields to certain liquids can lead to undesirable outcomes, such as gas bubble formation, pH fluctuations, or electrochemical decomposition. To address these challenges, several innovative designs and analytical models have been proposed. Brask et al. [13] introduced an EO pump that can move nonconducting liquids through viscous drag between two liquids. The innovation opens up new avenues for micro-Total Analysis Systems (μTAS) in pharmaceutical and environmental monitoring. Afonso et al. [14] developed an analytical model that describes a two-fluid EOF, demonstrating a pump concept where a nonconducting fluid is transported by the EOF of a conducting fluid through interfacial viscous drag forces. Daghighi et al. [15] observed vortices in electrokinetic flow around a conducting surface under a DC electric field, revealing differences in velocity between metal and nonconducting polymer particles of similar size. Barman and Bhattacharyya [16] conducted numerical simulations on the electrophoresis of nonconducting droplets in a hydrogel medium, showcasing the potential for size-based sorting through the manipulation of gel properties. Gao et al. [17,18] studied EO pumping and pressure-driven flow in microchannels involving two fluids, demonstrating precise control of the fluid interface position through adjustments to the electric field. Alyousef et al. [19] investigated the application of EO pumps in the fabrication of large implants. Moghadam and Akbarzadeh [20] studied the time-periodic behavior of two-liquid flow in microchannels, emphasizing the significance of various parameters on flow dynamics. Gaikwad et al. [21] examined the transport of immiscible fluid layers in a microfluidic channel under the combined influences of pressure and an electric field, highlighting the interaction between interfacial slip and electrical double layer (EDL) effects on flow dynamics. Deng and Xiao [22] investigate the transient two-layer EOF and heat transfer of power-law nanofluids in a microchannel, focusing on the effects of various parameters such as nanoparticle volume fraction and electrokinetic width on flow dynamics and thermal performance. This understanding is crucial for designing biomedical and biochemical microfluidic devices.

The aforementioned studies primarily focus on EOF in microchannels with smooth walls. However, wall roughness can arise due to the manufacturing process or the deposition of substances (such as macromolecules) on the wall. In some instances, artificially designed wall roughness can enhance the mixing efficiency of fluid systems. While microfluidic flow is typically laminar, an increase in relative wall roughness (compared to the channel radius) may introduce small disturbances into the mainstream region, thereby affecting the overall flow. These disturbances can impact component separation efficiency, mixing reactions, flow rates, and heat transfer processes within microfluidic systems. The impact of wall roughness on flow is a multifaceted issue, presenting both advantages and disadvantages.

Currently, most research is directed towards EOF in smooth microchannels [3,4,5,6,7,8,9,10,11,12,13,14,15,16,17,18,19,20,21,22], with relatively few studies examining the effects of wall roughness. Since the 1970s, several scholars have investigated laminar flow in rough-walled channels. Wang [23] was the first to study Stokes flow between flat plates with corrugated roughness. Chu [24] employed the perturbation expansion method to assess the impact of corrugation roughness on fluid flow. Xia et al. [25] analytically solved for EOF in a parallel plate microchannel, where one wall was smooth and the other exhibited sinusoidal corrugation, using the complex potential function and boundary integral method. They also analyzed the influence of corrugation amplitude and plate spacing on the flow field. Cho et al. [26,27,28] utilized the finite volume method (FVM) to numerically investigate the effects of wall corrugation, composed of two sinusoidal superpositions, on DC/AC EOF of Newtonian and power-law fluids between parallel plates. In special cases, when simplified to a single sinusoidal function to mimic corrugation, their results were consistent with those of Xia et al. [25]. Yoshida et al. [29] studied EOF in a narrow channel between corrugated walls using the lattice Boltzmann method (LBM) and analytical models. They observed that variations in channel width led to decreased flow velocity and non-uniform flow in the presence of an inhomogeneous surface charge distribution.

The Boundary Perturbation Method (BPM) has been extensively employed to investigate the EOF problem in microchannels characterized by corrugated walls. For example, Shu et al. [30] applied BPM to obtain an analytical solution for EOF in a parallel plate microchannel with longitudinal sinusoidal corrugation boundaries, validating its accuracy. Chang et al. [31] utilized BPM to study EOF in circular microfluidic channels featuring axial sinusoidal corrugation, considering the effects of relative corrugation amplitude, wave number, and pressure gradient on electric potential and velocity distributions. They elucidated the reasons behind the increase or decrease in velocity. Keramati et al. [32], in their work on circular microtubes, found that corrugated roughness adversely impacts EOF and heat transfer. Messinger and Squires [33] discovered that nanoscale wall roughness on micromachined metal electrodes can significantly suppress EOF when wall conductivity is high. Fakhari and Mirbozorg [34] conducted numerical studies using the FVM to assess the influence of various wall roughness types (sinusoidal, sawtooth, and square tooth) on EOF between parallel plates, concluding that wall roughness diminishes EOF velocity. Chang et al. [35] discussed the impact of sinusoidal roughness on AC EOF of Maxwell fluids in parallel microchannels. Mehta et al. [36] investigate the energy production assessment for heat flow of non-Newtonian ionic liquids within a wavy microchannel, focusing on the impacts of finite ionic size, electroosmotic actuation, and various parameters on entropy generation. Their findings have implications for the development of efficient heat-exchanging devices for electronic cooling. Nayak and Weigand [37] conducted a numerical analysis of fluid transport and mixing in micro/nano-channels with charged block walls, considering Joule heating, pressure variation, and electromigration. They revealed complex flow structures and enhanced mixing rates due to wall heterogeneity and improved ion transport. Xie et al. [38] further explored electrokinetic flow in a nanochannel with charged symmetric corrugated surfaces, studying the impact of corrugation geometry on flow characteristics, streaming potential, and energy conversion efficiency. They identified optimal corrugation parameters that enhance the streaming current and conversion efficiency in microfluidic devices. Maher et al. [39] investigated the effects of dusty fluids containing suspended solid particles in a single-walled corrugated channel using electromagnetic hydrodynamics, revealing the influence of corrugation on fluid and particle velocity behavior. They proposed a mathematical induction model for fluid control during curing stages, with potential applications in sanitation, drainage, and irrigation systems. Das et al. [40] presented a theoretical model to simulate the mixed convective flow of an ionic ternary hybrid nanofluid, driven by electroosmosis and magnetohydrodynamics, in a vertical nonconducting channel. They examined the impacts of various parameters on flow characteristics and proposed potential applications in electromechanical and nanofluidic devices. The influence of small-amplitude random lateral wall roughness on electro-magnetohydrodynamic (EMHD) in both parallel plate [41] and cylindrical [42] microchannels was studied through the perturbation method of stationary random function theory. Zhu et al. [43] numerically investigated flow and heat transfer characteristics in a microchannel with gradually expanding and suddenly contracting cross-sections, comparing it with microchannels of similar volume and nearly equivalent convective heat transfer area. Mohammadi et al. [44] utilized the FVM method to study the impact of nanofluids as coolants and sinusoidal walls on the performance of rectangular microchannel heat sinks. Qing et al. [45] investigated EOF and mass transfer of a Newtonian fluid driven by pressure gradients and AC electric fields in a parallel microchannel featuring sinusoidal roughness and modulated charged surfaces.

The impact of wall roughness or corrugation on the electroosmotic flow (EOF) in microchannels is a hot topic in current research. Early studies mainly focused on the flow characteristics of EOF in parallel plate or rectangular microchannels with wall roughness or corrugation, with research methods often analytical but not comprehensive. Some scholars have used micro-particle imaging velocimetry and laser Doppler anemometry [46,47,48,49] to measure the average velocity of EOF in rough microchannels. However, the widespread development of experimental studies is constrained by high technical requirements and costs of sample preparation, as well as long cycles.

In summary, most of the existing literature has concentrated on the corrugated wall effect in monolayer fluids, whereas the EOF of two-layer fluids within microchannels featuring sinusoidal corrugations has garnered insufficient attention. Notably, theoretical investigations and numerical simulations concerning two-layer EOF in rough channels are still in their nascent stages, highlighting the pressing need for comprehensive and mechanistic fundamental studies in this domain. Given this backdrop, the present paper employs the linearized Poisson–Boltzmann (P–B) equation and Navier–Stokes (N–S) equation to explore the impact of sinusoidal corrugations on the EOF of two-layer Newtonian fluids. This research endeavor is pivotal for a profound understanding of the behavior of conductive and insulating fluids within microchannels, as well as for the design and optimization of microfluidic systems.

## 2. Flow Geometry and Definitions

**Figure 1:**
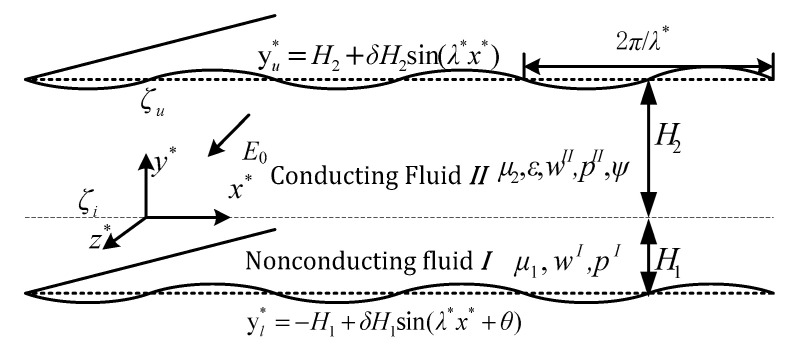
Schematic of EOF of two-layer fluid system through a microchannel with sinusoidal corrugation walls.

The flow studied in this section is a steady, fully developed flow of two incompressible and immiscible Newtonian fluids with differing conductivities. The nonconductive fluid occupies the lower section of the system and is propelled by the adjacent conductive fluid located in the upper layer, as shown in Figure 1. Although the coordinate system is positioned in the interface that delineates the two fluids, their respective thicknesses are not uniform.

The pressure gradient between the inlet and outlet of the upper and lower channels can be independently controlled and reflected in the same or opposite direction as the electric field. To analyze this system, a three-dimensional (3D) Cartesian orthogonal coordinate system (x*, y*, z*) is established, with the origin at the fluid–fluid interface. It is assumed that the average heights of the lower nonconductive fluid (fluid *I*) and the upper conducting fluid (fluid *II*) in the microchannel are *H*_1_ and *H*_2_ respectively. The length and width of the microchannel are significantly greater than its height *H*_1_ + *H*_2_, as shown in Figure 1.

An EDL forms naturally due to the interaction between the upper wall and the conducting fluid when a DC electric field of strength *E*_0_ is applied along the z* direction. This electric field exerts a Coulomb force on the ions within the fluid, initiating the generation of EOF by entraining fluid molecules in the direction of the field gradient. At the fluid–fluid interface, another EDL forms in the conducting fluid adjacent to the interface as a result of dielectric interactions. The conductive fluid (fluid *II*) moves under the influence of the Coulomb force and, through viscous shear stress, drags the underlying nonconductive fluid (fluid *I*), generating the overall flow.

The corrugated wall surfaces of the lower and upper plates are described by the equations y_l_* = *H*_1_ [−1 + *δ*sin(λ*x* + *θ*)] and y_u_* = *H*_2_ [1 + *δ*sin(λ*x*)], respectively. The EDL formed near the wall of the top channel in contact with fluid *II* exhibits a zeta potential *ζ_u_.* Additionally, the second EDL in fluid *II*, located at the interface with fluid *I*, has an interface zeta potential *ζ_i_.* This interfacial zeta potential is related to the properties of the two fluids, such as the pH value of the electrolyte solution, the ion concentration in the conductive fluid, and the presence of ionic surfactant. This interfacial zeta potential influences potential distribution within the two EDLs and thus the EO force and velocity distributions.

## 3. Mathematical Models and Approximate Solutions

The primary simplifying assumptions and considerations employed in the current analysis are described as follows: (i) both fluids are presumed to be viscous Newtonian fluids, exhibiting distinct conductivities; (ii) the properties of the fluids are deemed independent of the local electric field, ion concentration, and temperature (this assumption holds true for the scenario addressed in this paper); (iii) given the no-slip boundary condition on the channel wall, flow is assumed to be steady and fully developed; (iv) the two fluids are immiscible, leading to the formation of a planar interface that delineates their boundary. At this interface, the second EDL forms; (v) pressure gradients can be applied concurrently along the channel; (vi) the conditions stipulated by the classical electrodynamics theory are applicable in this study [50].

For conductive fluid (fluid *II*), considering Debye–Hückel linearization, the linearized P–B equation can be obtained:

(1) $$\frac{\partial^{2}\Psi }{\partial x^{*2}}+\frac{\partial^{2}\Psi }{\partial y^{*2}}=\kappa^{2}\Psi ,$$

where *κ* = *z_v_e*(2*n*_0_/*εk_b_T*)^1/2^ is the Debye–Hückel parameter and 1/*κ* represents the thickness of the EDL, i.e., the Debye length. The corresponding boundary condition is

(2) $$\Psi (x^{*},y^{*})=\zeta_{u},\;\mathrm{at}y=y_{u}^{*},\;\Psi (x^{*},y^{*})=\zeta_{i},\;\mathrm{at}y=0,$$

It is assumed here that the zeta potentials, *ζ_u_* and *ζ_i_* at the upper wall and the interface, respectively, remain constant [30].

For the incompressible nonconductive fluid (fluid *I*) and conductive fluid (fluid *II*), in which both fluid layers satisfy the continuity equation and N-S equation, the convective term in the N–S equation can be neglected, resulting in simplified governing equations:

(3) $$-\frac{\partial P^{i}}{\partial z^{*}}+\mu_{i}\nabla^{*2}W^{i}-\frac{2n_{0}z_{v}^{2}e^{2}}{k_{b}T}E_{0}\Psi \delta_{i2}=0,\;i=1,2,$$

where *z_ν_*, *e*, *n*_0_, *ε*, *k_b_*, *T*, *P^i^*, *μ_i_*_,_ and *δ_i_*_2_ respectively represent the valence of ions, the elementary charge, the number density of ions, the dielectric constant of electrolyte solution, the Boltzmann constant, the absolute temperature, the pressure of the i-th layer fluid, the dynamic viscosity of the i-th layer (where *i* = 1 for layer *I*, *i* = 2 for layer *II*) and the Kronecker tensor.

Velocity satisfies the no-slip condition at the channel wall and the continuity of shear stress at the interface between the two fluids:

(4) $$\begin{array}{l} \;\;\{\begin{array}{l} W^{II}(x^{*},y^{*})=0,\mathrm{at}\;y=y_{u}^{*},\\ W^{I}(x^{*},y^{*})=0,\mathrm{at}\;y=y_{l}^{*},\\ W^{I}(x^{*},y^{*})=W^{II}(x^{*},y^{*}),\mu_{1}\frac{\partial W^{I}(x^{*},y^{*})}{\partial y^{*}}=\mu_{2}\frac{\partial W^{II}(x^{*},y^{*})}{\partial y^{*}},\mathrm{at}\;y=0. \end{array}\\ \mathrm{Introduce}\;a\;\mathrm{set}\;\mathrm{of}\;\mathrm{dimensionless}\;\mathrm{parameters}: \end{array}$$

(5) $$(x,y)=\frac{\left(x^{*},y^{*}\right)}{H_{2}},\;K=\kappa H_{2},\;\varphi \left(x,y\right)=\frac{\Psi \left(x^{*},y^{*}\right)}{\zeta_{u}},\;w^{i}\left(x,y\right)=\frac{W^{i}(x^{*},y^{*})}{U_{eo}},\;U_{eo}=-\frac{\varepsilon \zeta_{u}E_{0}}{\mu_{2}},G^{i}=-\frac{H_{2}^{2}}{\mu_{i}U_{eo}}\frac{\partial P^{i}}{\partial z^{*}},\zeta =\frac{\zeta_{i}}{\zeta_{u}},h_{r}=\frac{H_{1}}{H_{2}},\mu_{r}=\frac{\mu_{1}}{\mu_{2}},$$

In the above formula, the dimensionless electrokinetic width *K* represents the ratio of the average height of fluid *II*(*H*_2_) to the Debye length (1/*κ*); *G^i^* represents the dimensionless pressure gradient exerted in the axial direction of the channel.

By substituting Equation (5) into Equations (1)–(4), we obtain dimensionless governing equations and boundary conditions:

(6) $$\frac{\partial^{2}\varphi }{\partial x^{2}}+\frac{\partial^{2}\varphi }{\partial y^{2}}=K^{2}\varphi ,$$

(7) $$\frac{\partial^{2}w^{i}}{\partial x^{2}}+\frac{\partial^{2}w^{i}}{\partial y^{2}}=-G^{i}-\frac{\mu_{i}}{\mu_{2}}K^{2}\varphi \delta_{i2}.$$

The corresponding boundary conditions are

(8) $$\left\{\begin{array}{l} \varphi \left(x,y\right)=1,w^{II}\left(x,y\right)=0,\mathrm{at}\;y=y_{u},\\ \varphi \left(x,y\right)=\zeta ,w^{II}\left(x,y\right)=w^{I}\left(x,y\right),\mu_{r}\frac{\partial w^{I}(x,y)}{\partial y}=\frac{\partial w^{II}(x,y)}{\partial y},\mathrm{at}\;y=0,\\ \;w^{I}\left(x,y\right)=0,\mathrm{at}\;y=y_{l}. \end{array}\right.$$

Assuming *δ* << 1, the electric potential *φ* and velocity *w*^i^ can be expanded in power series:

(9) $$R\left(x,y\right)=R_{0}\left(y\right)+\delta R_{1}\left(x,y\right)+\delta^{2}R_{2}\left(x,y\right)+\cdots ,$$

On the upper wall surface at *y = y_u_* and the lower wall surface at *y = y_l_*, the Taylor expansion of the function *R* is considered at *y* = 1 and *y =* −*h_r_*, respectively:

(10) $$\left\{\begin{array}{l} R\left(x,1+\delta \sin(\lambda x)\right)=R\left(x,1\right)+\delta \sin(\lambda x)R_{y}\left(x,1\right)+\frac{\delta^{2}\sin^{2}(\lambda x)}{2}R_{yy}\left(x,1\right)+\cdots \\ \;\;\;\;\;\;\;=R_{0}\left(1\right)+\delta [\sin\left(\lambda \theta \right)R_{0}^{\prime }\left(1\right)+R_{1}\left(x,1\right)]+\delta^{2}[\frac{\sin^{2}\left(\lambda \theta \right)}{2}R_{0}^{\prime \prime }\left(1\right)+\sin\left(\lambda \theta \right)R_{1y}\left(x,1\right)+R_{2}\left(x,1\right)]+\cdots \\ R\left(x,-h_{r}+\delta h_{r}\sin(\lambda x+\theta )\right)=R\left(x,-h_{r}\right)+\delta h_{r}\sin(\lambda x+\theta )R_{y}\left(x,-h_{r}\right)+\frac{\delta^{2}h_{r}^{2}\sin^{2}(\lambda x+\theta )}{2}R_{yy}\left(x,-h_{r}\right)+\cdots \\ \;\;\;\;\;=R_{0}\left(-h_{r}\right)+\delta [h_{r}\sin\left(\lambda x+\theta \right)R_{0}^{\prime }\left(-h_{r}\right)+R_{1}\left(x,-h_{r}\right)]+\delta^{2}[\frac{h_{r}^{2}\sin^{2}\left(\lambda \theta +\theta \right)}{2}R_{0}^{\prime \prime }\left(-h_{r}\right)+\\ \;\;\;\;\;\;h_{r}\sin\left(\lambda x+\theta \right)R_{1y}\left(x,-h_{r}\right)+R_{2}\left(x,-h_{r}\right)]+\cdots . \end{array}\right.$$

Substituting Equation (9) into Equations (6) and (7), and equating coefficients of *δ*, we obtain

(11) $$\delta^{0}:\;\frac{d^{2}\varphi_{0}}{dy^{2}}=K^{2}\varphi_{0},\frac{d^{2}w_{0}^{II}}{dy^{2}}=-G^{II}-K^{2}\varphi_{0}^{II},\frac{d^{2}w_{0}^{I}}{dy^{2}}=-G^{I},$$

(12) $$\delta^{1}:\;\frac{\partial^{2}\varphi_{1}}{\partial x^{2}}+\frac{\partial^{2}\varphi_{1}}{\partial y^{2}}=K^{2}\varphi_{1},\frac{\partial^{2}w_{1}^{II}}{\partial x^{2}}+\frac{\partial^{2}w_{1}^{II}}{\partial y^{2}}=-K^{2}\varphi_{1},\;\frac{\partial^{2}w_{1}^{I}}{\partial x^{2}}+\frac{\partial^{2}w_{1}^{I}}{\partial y^{2}}=0,$$

(13) $$\delta^{2}:\;\frac{\partial^{2}\varphi_{2}}{\partial x^{2}}+\frac{\partial^{2}\varphi_{2}}{\partial y^{2}}=K^{2}\varphi_{2},\frac{\partial^{2}w_{2}^{II}}{\partial x^{2}}+\frac{\partial^{2}w_{2}^{II}}{\partial y^{2}}=-K^{2}\varphi_{2},\frac{\partial^{2}w_{2}^{I}}{\partial x^{2}}+\frac{\partial^{2}w_{2}^{I}}{\partial y^{2}}=0.$$

Using the Taylor expansion (10), the corresponding boundary conditions for Equations (11)–(13) can be obtained from Equation (8) as

(14) $$\delta^{0}:\varphi_{0}\left(1\right)=1,\;w_{0}^{II}\left(1\right)=0,\;w_{0}^{I}\left(-h_{r}\right)=0,\varphi_{0}\left(0\right)=\zeta ,\;w_{0}^{II}\left(0\right)=w_{0}^{I}\left(0\right),\frac{dw_{0}^{II}}{dy}{|}_{y=0}=\mu_{r}\frac{dw_{0}^{I}}{dy}{|}_{y=0},$$

(15) $$\delta^{1}:\varphi_{1}\left(x,1\right)=-\sin(\lambda x)\varphi_{0}^{\prime }\left(1\right),\;\varphi_{1}\left(x,0\right)=0,w_{1}^{II}\left(x,0\right)=w_{1}^{I}\left(x,0\right),\;\frac{\partial w_{1}^{II}}{\partial y}{|}_{y=0}=\mu_{r}\frac{\partial w_{1}^{I}}{\partial y}{|}_{y=0},\;w_{1}^{II}\left(x,1\right)=-\sin(\lambda x)\frac{dw_{0}^{II}}{dy}{|}_{y=1},\;w_{1}^{I}\left(x,-h_{r}\right)=-h_{r}\sin(\lambda x+\theta )\frac{dw_{0}^{I}}{dy}{|}_{y=-h_{r}},$$

(16) $$\delta^{2}:\;\varphi_{2}\left(x,1\right)=-\frac{\sin^{2}\left(\lambda x\right)}{2}\varphi_{0}^{\prime \prime }\left(1\right)-\sin\left(\lambda x\right)\frac{\partial \varphi_{1}}{\partial y}{|}_{y=1},\varphi_{2}\left(x,0\right)=0,w_{2}^{II}\left(x,0\right)=w_{2}^{I}\left(x,0\right),\;\frac{\partial w_{2}^{II}}{\partial y}{|}_{y=0}=\mu_{r}\frac{\partial w_{2}^{I}}{\partial y}{|}_{y=0},\;w_{2}^{I}\left(x,-h_{r}\right)=-\frac{h_{r}^{2}\sin^{2}\left(\lambda x+\theta \right)}{2}\frac{d^{2}w_{0}^{I}}{dy^{2}}{|}_{y=-h_{r}}-h_{r}\sin\left(\lambda x+\theta \right)\frac{\partial w_{1}^{I}}{\partial y}{|}_{y=-h_{r}}.$$

The general solution to Equation (11) is

(17) $$\varphi_{0}(y)=A_{1}\cosh\left(Ky\right)+A_{2}\sinh(Ky),w_{0}^{II}\left(y\right)=C_{1}+C_{2}y-\frac{G^{II}}{2}y^{2}-A_{1}\cosh\left(Ky\right)-A_{2}\sinh(Ky),w_{0}^{I}\left(y\right)=D_{1}+D_{2}y-\frac{G^{I}}{2}y^{2}.$$

Substituting Equation (14) into Equation (17), the undetermined constants *A_j_*, *C_j_*, *D_j_*, (*j* = 1, 2) can be obtained, as shown in Appendix A.

According to the boundary condition (15), the solution form of Equation (12) can be expressed as

(18) $$\varphi_{1}\left(x,y\right)=\;f_{1}(y)\sin\left(\lambda x\right),w_{1}^{II}\left(x,y\right)=\;F_{1}(y)\sin\left(\lambda x\right)+F_{2}(y)cos(\lambda x),\;w_{1}^{I}\left(x,y\right)=\;G_{1}(y)\sin\left(\lambda x\right)+G_{2}(y)\cos(\lambda x).$$

Substituting Equation (18) into Equation (12), we separate and organize the calculation to get

(19) $$f_{1}\left(y\right)=A_{3}\cosh(K_{1}y)+A_{4}\sinh(K_{1}y),\;F_{1}\left(y\right)=C_{3}\cosh\lambda y+C_{4}\sinh\lambda y-A_{3}\cosh(K_{1}y)-A_{4}\sinh(K_{1}y),\;F_{2}\left(y\right)=C_{5}\cosh\lambda y+C_{6}\sinh\lambda y,\;G_{1}\left(y\right)=D_{3}\cosh\lambda y+D_{4}\sinh\lambda y,G_{2}\left(y\right)=D_{5}\cosh\lambda y+D_{6}\sinh\lambda y,$$

where $K_{1}^{2}=K^{2}+\lambda^{2}$ and the undetermined constants are *A_j_*, *C_k_*, *D_k_* (*j* = 3, 4; *k* = 3, 4, 5, 6), as shown in Appendix A.

According to the boundary condition (16), the solutions of Equation (13) can be expressed in the form

(20) $$\varphi_{2}\left(x,y\right)=\;f_{2}(y)+f_{3}(y)\sin\left(2\lambda x\right),w_{2}^{II}\left(x,y\right)=F_{3}\left(y\right)+\;F_{4}(y)\sin\left(2\lambda x\right)+F_{5}(y)\cos(2\lambda x),\;w_{2}^{I}\left(x,y\right)=G_{3}\left(y\right)+\;G_{4}(y)\sin\left(2\lambda x\right)+G_{5}(y)\cos(2\lambda x).$$

Similarly, substituting Equation (20) into Equation (13), we separate and rearrange it to obtain

(21) $$f_{2}\left(y\right)=A_{5}\cosh\left(Ky\right)+A_{6}\sinh(Ky),\;f_{3}\left(y\right)=A_{7}\cosh\left(K_{2}y\right)+A_{8}\sinh(K_{2}y),\;F_{3}\left(y\right)=C_{7}+C_{8}y-A_{5}\cosh\left(Ky\right)-A_{6}\sinh(Ky),\;F_{4}\left(y\right)=C_{9}\cosh(2\lambda y)+C_{10}\sinh(2\lambda y),\;F_{5}\left(y\right)=C_{11}\cosh\left(2\lambda y\right)+C_{12}\sinh\left(2\lambda y\right)-A_{7}\cosh\left(K_{2}y\right)-A_{8}\sinh(K_{2}y),\;G_{3}\left(y\right)=D_{7}+D_{8}y,\;G_{4}\left(y\right)=D_{9}\cosh(2\lambda y)+D_{10}\sinh(2\lambda y),\;G_{5}\left(y\right)=D_{11}\cosh\left(2\lambda y\right)+D_{12}\sinh\left(2\lambda y\right),$$

where $K_{2}^{2}=K^{2}+4\lambda^{2}$ and the undetermined constants are *A_j_*, *C_k_*, *D_k_* (*j* = 5, 6, 7, 8; *k* = 7, 8, …, 12), as shown in Appendix A.

## 4. Average Velocity

By averaging the flow rate per unit width of the microchannel over one wavelength of the corrugated wall, the average velocity of the upper fluid *II* can be derived:

(22) $${\bar{u}}^{II}=\frac{\lambda }{2\pi }\int_{0}^{\frac{2\pi }{\lambda }}dx\int_{0}^{1+\delta \sin(\lambda x)}w^{II}\left(x,y\right)dy=u_{0m}^{II}+\delta^{2}u_{2m}^{II}+O(\delta^{4}),$$

where

(23) $$u_{0m}^{II}=C_{1}+\frac{C_{2}}{2}-\frac{G^{II}}{6}-\frac{\left(\cosh K-1\right)A_{2}+A_{1}\sinh K}{K},u_{2m}^{II}=C_{7}+\frac{1}{2}C_{8}+\frac{(1-\cosh K)A_{6}}{K}+\frac{1}{4}\frac{dW_{0}^{II}}{dy}{|}_{y=1}.$$

Similarly, by averaging the flow rate per unit width of the microchannel over a wavelength of the corrugated wall, the average velocity of the lower fluid *I* can be derived:

(24) $${\bar{u}}^{I}=\frac{\lambda }{2\pi h_{r}}\int_{0}^{\frac{2\pi }{\lambda }}dx\int_{-h_{r}+\delta h_{r}\sin(\lambda x+\theta )}^{0}w^{I}\left(x,y\right)dy=u_{0m}^{I}+\delta^{2}u_{2m}^{I}+O(\delta^{4}),$$

where

(25) $$u_{0m}^{I}=D_{1}-\frac{h_{r}}{6}\left(3D_{2}+G^{I}h_{r}\right),u_{2m}^{I}=D_{7}-\frac{h_{r}}{2}D_{8}+\frac{1}{2}G_{2}\left(-h_{r}\right)\sin\theta +\frac{1}{2}G_{1}\left(-h_{r}\right)\cos\theta +\frac{h_{r}}{4}\frac{dW_{0}^{I}}{dy}{|}_{y=-h_{r}}.$$

## 5. Results and Discussion

Previous studies present an approximate analytical solution of velocity for the mixed EO pressure-driven flow of a two-layer system comprising Newtonian fluids within a microchannel with sinusoidal corrugations. It mainly depends on the electrokinetic width *K*, the zeta potential ratio *ζ* between the fluid–fluid interface and the upper wall surface, the corrugation wave number *λ*, the phase difference *θ* between the corrugations on the upper and lower walls, the dimensionless pressure gradient *G* (assuming *G^I^* = *G^II^* = 0 for simplicity), the depth ratio of the bottom to top fluid *h_r_*, the viscosity ratio of the bottom to top fluid *μ_r_*, and the ratio of the corrugation amplitude to the average height of the top fluid layer fluid *δ*. In the following calculations, the typical parameter δ is limited to *δ* < 0.1 to ensure the validity of our sinusoidal approximation. The characteristic scale of the upper layer microchannel is *H*_2_ = 100 μm and the viscosity of the upper layer fluid is *μ*_2_ = 10^−3^ kg/ms. Unless specified otherwise, the default parameter values utilized in this section are as follows: *K* = 10, *h_r_* = 0.5, *λ* = 8, and *G* = 0 (implying *G^I^ = G^II^* = 0).

**Figure 2:**
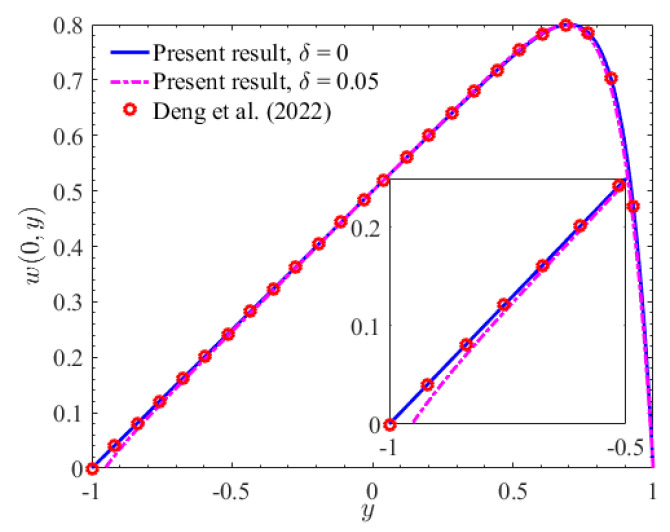
The variation of velocity *w*(0, *y*) with *y* (*ζ* = 0, *h_r_* =1) [22].

With *x* = 0, Figure 2 depicts the velocity amplitude profile of the two-layer Newtonian fluid within the microchannel. It is evident that the velocity amplitude in the rough microchannel is notably smaller than that in the smooth microchannel. This observation can be attributed to the increased contact area between the fluid and the corrugation on the lower wall of the rough microchannel. This enhancement in contact area leads to a corresponding increase in flow resistance, ultimately resulting in a reduction of velocity amplitude. Furthermore, in the smooth microchannel, the velocity profile is consistent with the findings reported by Deng et al. [22].

**Figure 3:**
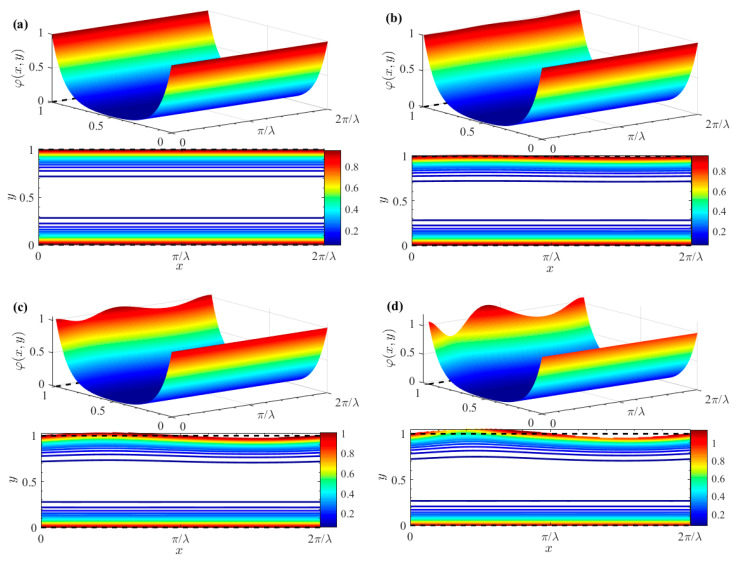
3D zeta potential distributions and contours for different *δ* (*ζ* = 1). (**a**) *δ* = 0; (**b**) *δ* = 0.01; (**c**) *δ* = 0.03; (**d**) *δ* = 0.05.

Figure 3 shows the distribution of the dimensionless potentials for various values of *δ*. Specifically, the potential distribution in the case of a smooth channel (where *δ* = 0) is depicted in Figure 3a. It becomes evident that the electric potential fluctuation near the wall becomes increasingly significant as the corrugation amplitude *δ* increases. The observation highlights the significant influence of surface roughness on the electric potential distribution. Furthermore, the figure reveals that electric potential is particularly high within the EDL adjacent to the wall. In contrast, the potential undergoes a sharp decline in the narrow region outside the EDL. The sharp drop in potential can be attributed to the rapid transition from the highly charged EDL to the relatively uncharged bulk fluid.

**Figure 4:**
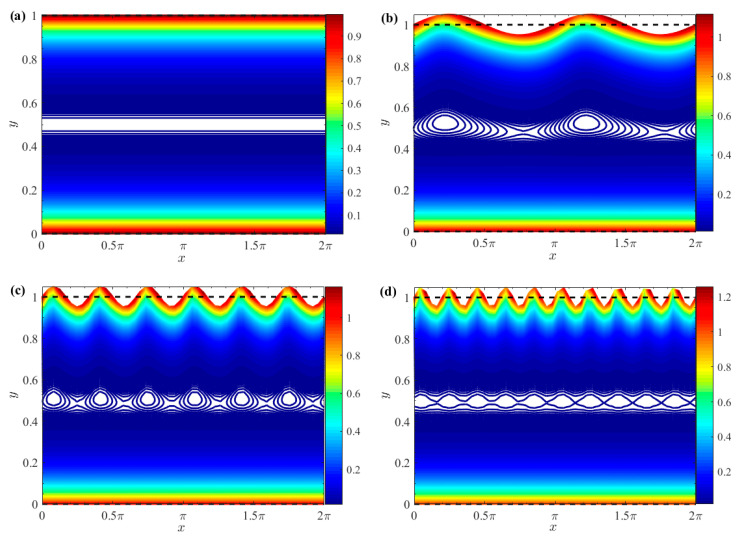
Zeta potential contours for different *δ* and *λ* (*ζ* = 1). (**a**) *λ* = 2, *δ* = 0; (**b**) *λ*= 2, *δ* = 0.05; (**c**) *λ* = 6, *δ* = 0.05; (**d**) *λ* = 10, *δ* = 0.05.

Figure 4 shows the effects of varying corrugation amplitude δ and wave numbers λ on electric potential distribution within the EDL along the wall. For smooth walls (*δ* = 0), EDL electric potential is smooth and uniform, as shown in Figure 4a. In contrast, Figure 4b–d demonstrate the effect of a finite wall corrugation with *δ* set to 0.05. As wall corrugation increases, it enhances resistance to fluid flow. This increased resistance influences EDL electric potential, causing it to oscillate in response to the sinusoidal wall undulations. Moreover, higher wave numbers, associated with shorter wavelengths, lead to more frequent fluctuations. Consequently, EDL electric potential exhibits more pronounced periodic oscillations near the wall. These oscillations induce additional disturbances, further affecting the overall electric potential distribution.

**Figure 5:**
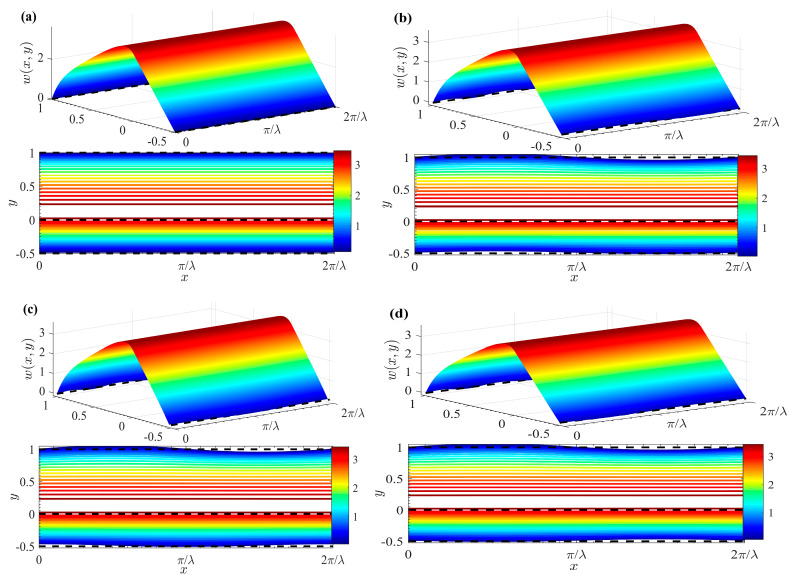
3D velocity distributions and contours for different *δ* and *θ* (*μ_r_* = 1, *ζ* = 1). (**a**) *δ* = 0; (**b**) *θ* = 0, *δ* = 0.05; (**c**)*θ* = *π*/2, *δ* = 0.05; (**d**) *θ* = *π*, *δ* = 0.05.

Figure 5 draws the 3D velocity and contour distribution of two-layer Newtonian fluids under different *δ* and *θ*. Notably, the velocity distribution is significantly influenced by *θ*. Furthermore, the flow of fluid *II*, as demonstrated in Figure 5, is exclusively driven by the EO force under an external electric field. When the EO force is applied within the EDL, fluid *II* achieves peak velocity at the fluid–fluid interface. Moreover, the flow of fluid *I* arises from the viscous shear stress at the fluid–fluid interface, coupled with the drag imparted by the electric field force exerted by the top layer of fluid *II*. So, the velocity profile of fluid *I* exhibits a linear decrement as it moves away from the interface.

**Figure 6:**
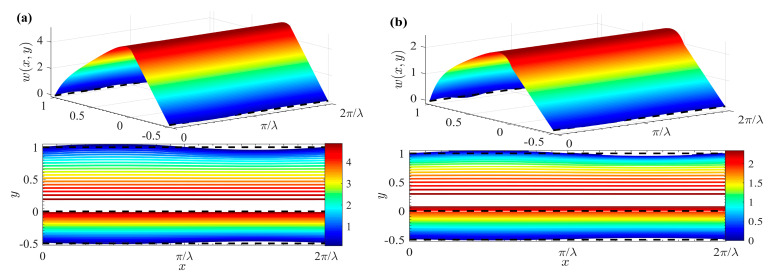
3D velocity distributions and contours for different *δ* and *θ* (*θ* = *π*, *δ* = 0.05, *ζ* = 1). (**a**) *μ_r_* = 0.5; (**b**) *μ_r_* = 2.

Figure 6 reveals the 3D velocity and contour distribution of two-layer Newtonian fluids at different *μ_r_*. By comparing Figure 6 with Figure 5d, it can be concluded that the velocity of the fluids decreases as *μ_r_* increases. The observation is intuitive, as fluid *I* with lower viscosity is expected to have the highest velocity. The reason for this is that the enlargement of *μ_r_* signifies an increase in the viscosity of fluid *I* relative to fluid *II*. Therefore, the viscous shear stress (or drag force) at the interface between the two fluids increases, leading to a reduction in the drag force exerted by fluid *II* on fluid *I*.

**Figure 7:**
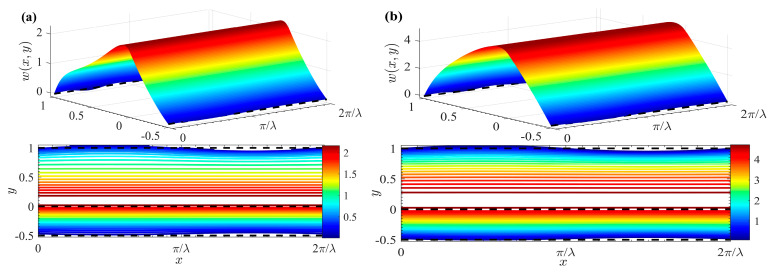
3D velocity distributions and contours for different *G* (*θ* = *π*, *δ* = 0.05, *ζ* = 1). (**a**) *G^I^ = G^II^* = −5; (**b**) *G^I^ = G^II^* = 5.

Figure 7 reflects the 3D velocity and contour distribution of a two-layer Newtonian fluid for various cases of *G^I^* and *G^II^*. A comparison between Figure 5d and Figure 7 reveals that fluid velocity increases with an increase of *G*. Specifically, a positive pressure gradient (*G^I^* > 0 and *G^II^* > 0) promotes fluid flow, whereas a negative pressure gradient (*G^I^* < 0 and *G^II^* < 0) hinders it.

**Figure 8:**
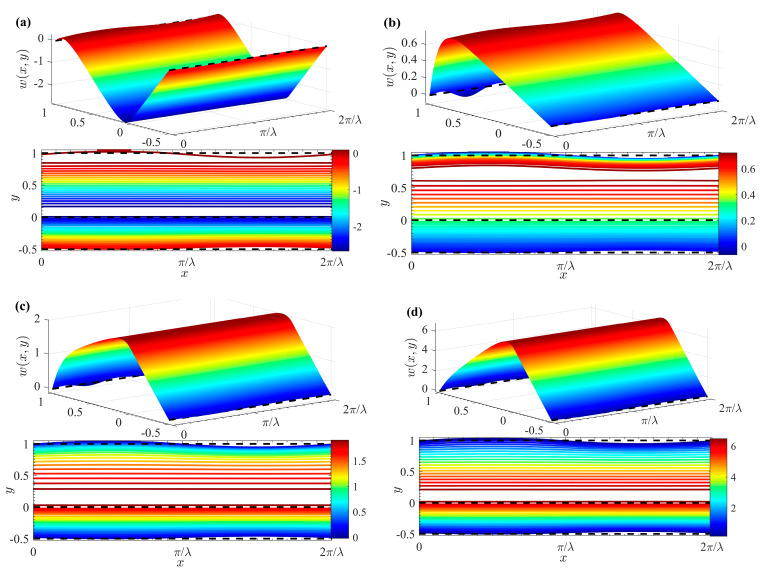
3D velocity distributions and contours for different *ζ* (*θ* = *π*, *δ* = 0.05). (**a**) *ζ* = -1; (**b**) *ζ* = 0; (**c**) *ζ* = 0.5; (**d**) *ζ* = 2.

Figure 8 shows the 3D velocity and contour distribution of two layers of Newtonian fluid under different *ζ*. By comparing Figure 5d and Figure 8, it can be seen that velocity escalates with the increment of ζ. At the interface between the two fluids, the velocity distribution undergoes a notable modification due to the influence of a favorable additional Coulomb force, resulting in a significant increase in velocity. Furthermore, when the fluid–fluid interface and the upper plate carry opposite charges (i.e., *ζ* < 0), the direction of the EOF in fluid *II* aligns directly with the charge polarity of the channel wall. This phenomenon aligns with previous conclusions on the behavior of EOF in such systems.

**Figure 9:**
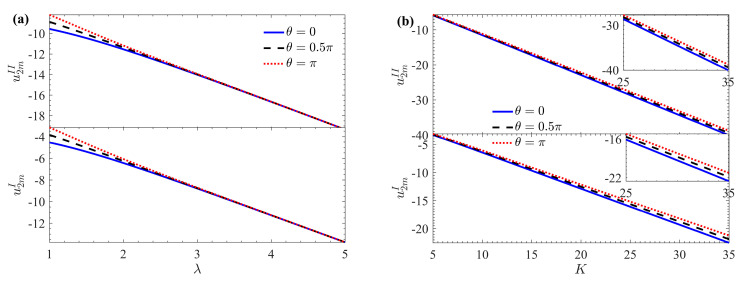

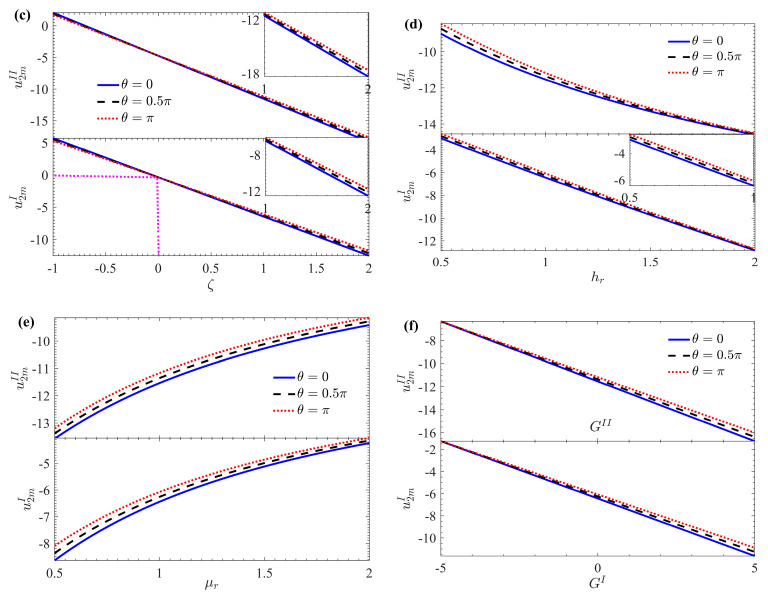
Variations of mean velocity increment *u*_2*m*_ with (**a**) *λ*; (**b**) *K*; (**c**) *ζ*; (**d**) *h_r_*; (**e**) *μ_r_*; (**f**) *G* for different *θ* at *K* = 10, *ζ* = 1, *λ* = 2, *δ* = 0.1, *h_r_* = 1, *G^II^* = *G^I^* =0, *μ_r_* = 1.

The variation of the average velocity increment (roughness function) *u*_2*m*_ is shown in Figure 9 with respect to *λ*, *K*, *ζ*, *h_r_*, *μ_r_*, and *G* under different *θ* (*ζ* = 1, *λ* = 2, *δ* = 0.1, *h_r_* = 1, *μ_r_* = 1). The *u*_2*m*_ decreases with increasing *λ*, *K*, *ζ*, *h_r_*_,_ and *G*. As expected, for larger wave numbers (such as *λ* > 2.8), the effect of *θ* on flow velocity becomes negligible. However, *u*_2*m*_ increases with an increase of *θ*, and the effect is more pronounced for smaller wave numbers (such as *λ* ≤ 2.8), aligning with previous findings [31]. A smaller *λ* corresponds to a larger wavelength, suggesting that in microchannels with long-wave rough corrugations, flow behavior can be approximated to that in a smooth microchannel (Figure 9a). When the EDL is extremely thin (indicated by a larger *K*), ions require only a small potential difference to reach the electrode surface, leading to a faster flow rate. However, fluid motion on the microchannel walls is influenced by corrugation roughness, resulting in increased flow drag. Therefore, *u*_2*m*_ decreases as *K* increases (Figure 9b). A significant impact on *u*_2*m*_ is observed due to the non-zero interface zeta potential, as shown in Figure 9c. When *ζ* > 0, a favorable additional Coulomb force emerges in the velocity distribution at the interface between the two fluids, resulting in a significant reduction of the roughness function *u*_2*m*_. When *ζ* < 0, unfavorable local electrostatic forces diminish the pumping effect, increasing the corresponding *u*_2*m*_. Notably, when *ζ* = 0, the roughness function *u*_2*m*_ of the fluid *I* is zero. As expected, a larger *h_r_* corresponds to an increase in the thickness of the bottom fluid *I* and a decrease in the thickness of the top fluid *II*, leading to a decrease in *u*_2*m*_ (Figure 9d). Additionally, *u*_2*m*_ increases as *μ_r_* increases. (Figure 9e). Here *μ_r_* represents the ratio of the viscosity of fluid *I* to that of fluid *II*. Therefore, the increase in *μ_r_* means that the viscosity of fluid *I* increases, the resistance of the viscous stress at the interface increases, and the drag force of fluid *II* on fluid *I* decreases. It is evident from Figure 9f that *u*_2*m*_ decreases as *G* increases. The dimensionless pressure gradient either promotes (when *G^I^* > 0 and *G^II^* > 0) or hinders (when *G^I^* < 0 and *G^II^* < 0) fluid flow, resulting in *u*_2*m*_ having an opposite effect to velocity. Furthermore, Figure 9 demonstrates wall roughness corrugation has a lesser effect on *u^I^*_2*m*_ than *u^II^*_2*m*_. This is because the driving force for fluid *I* is generated by the drag from viscous shear stress at the interface, and the drag encountered by fluid *II* is smaller than that of fluid *I*.

## 6. Conclusions

To optimize the performance of two-layer EO pumping systems in microdevices, this study employs the perturbation expansion method and the principle of linear superposition to investigate the steady, fully developed flow of immiscible Newtonian fluids within a parallel plate microchannel with sinusoidal corrugations. We assume the presence of an upper conductive fluid (fluid *II*) and a lower nonconductive fluid (fluid *I*). Theoretical analysis and graphical illustrations yield the following conclusions:

- The velocity amplitude in rough microchannels is significantly lower than in smooth channels, due to increased contact area and subsequent flow resistance caused by wall undulations.
- The electric potential distribution is significantly influenced by surface roughness, with pronounced fluctuations observed near the corrugated walls. This highlights the substantial impact of microchannel wall topography on fluid dynamics and EDL interactions.
- The velocity distribution within the microchannel is notably influenced by the phase difference *θ* between the upper and lower wall surface roughness.
- In fluid *II*, velocity peaks at the fluid–fluid interface, while in fluid *I*, it exhibits a linear decrease.
- The increasing *μ_r_* leads to decreased velocity, while *G* and higher *ζ* enhance fluid flow.
- The average velocity increment (represented by the corrugated wall roughness function) *u*_2*m*_, decreases with *λ*, *K*, *ζ*, *h_r_* and *G*, but increases with *μ_r_*.

## Data Availability

The original contributions presented in the study are included in the article, further inquiries can be directed to the corresponding author.

## Appendix A

$$\begin{array}{l} A_{1}=\zeta ,A_{2}=\frac{1-\zeta \cosh(K)}{\sinh(K)},C_{1}=D_{1}+A_{1},C_{2}=\mu_{r}D_{2}+A_{2}K,\\ D_{1}=\frac{h_{r}(G^{II}+2A_{1}(\cosh K-1)+2A_{2}(\sinh K-K)+G^{I}h_{r}\mu_{r})}{2(h_{r}+\mu_{r})},D_{2}=\frac{D_{1}}{h_{r}}-\frac{G^{I}h_{r}}{2},A_{3}=0,\;\;A_{4}=\frac{f_{1}(1)}{\sinh(K_{1})},\\ C_{3}=\frac{A_{4}\sinh(\lambda h_{r})(\lambda \sinh K_{1}-K_{1}\sinh\lambda )+\lambda [\sinh\left(\lambda h_{r}\right)F_{1}\left(1\right)+\mu_{r}G_{1}\left(-h_{r}\right)\sinh\lambda ]}{\lambda [\sinh\left(\lambda h_{r}\right)\cosh\lambda +\mu_{r}\cosh\left(\lambda h_{r}\right)\sinh\left(\lambda \right)]},\\ D_{3}=C_{3},C_{4}=\frac{F_{1}(1)+A_{4}\sinh K_{1}-C_{3}\cosh\lambda }{\sinh\lambda },D_{4}=\frac{C_{4}\lambda -K_{1}A_{4}}{\lambda \mu_{r}},D_{5}=\frac{\mu_{r}G_{2}\left(-h_{r}\right)\sinh\lambda }{\sinh(\lambda h_{r})\cosh\lambda +\mu_{r}\cosh(\lambda h_{r})\sinh\lambda },C_{5}=D_{5},\\ D_{6}=\frac{D_{5}\cosh(\lambda h_{r})-G_{2}(-h_{r})}{\sinh(\lambda h_{r})},C_{6}=D_{6}\mu_{r},A_{5}=0,\;\;A_{6}=f_{2}\left(1\right)\csc hK,\;A_{7}=0,\;\;A_{8}=f_{3}(1)\csc hK_{2},\\ C_{7}=\frac{h_{r}A_{6}(\sinh K-K)+h_{r}F_{3}(1)+\mu_{r}G_{3}(-h_{r})}{h_{r}+\mu_{r}},\;D_{7}=C_{7},C_{8}=F_{3}(1)-C_{7}+A_{6}\sinh K,D_{8}=\frac{C_{8}-KA_{6}}{\mu_{r}},\\ C_{9}=\frac{\sinh(2\lambda h_{r})F_{4}(1)+\mu_{r}\sinh(2\lambda )G_{4}(-h_{r})}{\mu_{r}\cosh(2\lambda h_{r})\sinh(2\lambda )+\cosh(2\lambda )\sinh(2\lambda h_{r})},D_{9}=C_{9},\;C_{10}=\frac{F_{4}(1)-C_{9}\cosh(2\lambda )}{\sinh(2\lambda )},D_{10}=\frac{C_{10}}{\mu_{r}},\\ C_{11}=\frac{A_{8}\sinh(2\lambda h_{r})[2\lambda \sinh K_{2}-K_{2}\sinh\left(2\lambda \right)]+2\lambda [\sinh(2\lambda h_{r})F_{5}(1)+\mu_{r}\sinh(2\lambda )G_{5}(-h_{r})]}{2\lambda [\cosh\left(2\lambda \right)\sinh\left(2\lambda h_{r}\right)+\mu_{r}\cosh\left(2\lambda h_{r}\right)\sinh\left(2\lambda \right)]},\\ D_{11}=C_{11},C_{12}=\frac{F_{5}\left(1\right)+A_{8}\sinh K_{2}-C_{11}\cosh(2\lambda )}{\sinh(2\lambda )},D_{12}=\frac{2\lambda C_{12}-K_{2}A_{8}}{2\lambda \mu_{r}}, \end{array}$$

where $K_{1}^{2}=\lambda^{2}+K^{2}$, $K_{2}^{2}=4\lambda^{2}+K^{2}$, $f_{1}(1)=-\varphi_{0}^{\prime }(1)$, $F_{1}(1)=-\frac{dw_{0}^{II}}{dy}{|}_{y=1}$, $G_{1}\left(-h_{r}\right)=-h_{r}\cos\theta \frac{dw_{0}^{I}}{dy}{|}_{y=-h_{r}}$, $G_{2}\left(-h_{r}\right)=-h_{r}\sin\theta \frac{dw_{0}^{I}}{dy}{|}_{y=-h_{r}}$, $f_{2}(1)=-\frac{1}{4}\varphi_{0}^{\prime \prime }\left(1\right)-\frac{1}{2}f_{1}^{\prime }\left(1\right),f_{3}\left(1\right)=\frac{1}{4}\varphi_{0}^{\prime \prime }\left(1\right)+\frac{1}{2}f_{1}^{\prime }\left(1\right)$, $F_{3}(1)=-\frac{1}{4}\frac{dw_{0}^{II}}{dy}{|}_{y=1}-\frac{1}{2}F_{1}^{\prime }\left(1\right),\;F_{4}\left(1\right)=-\frac{1}{2}F_{2}^{\prime }\left(1\right)$, $F_{5}\left(1\right)=\frac{1}{4}\frac{dw_{0}^{II}}{dy}{|}_{y=1}+\frac{1}{2}F_{1}^{\prime }\left(1\right),$$G_{3}\left(-h_{r}\right)=-\frac{h_{r}}{2}[\cos\theta G_{1}^{\prime }\left(-h_{r}\right)+\sin\theta G_{2}^{\prime }\left(-h_{r}\right)]-\frac{h_{r}^{2}}{4}\frac{d^{2}w_{0}^{I}}{dy^{2}}{|}_{y=-h_{r}}$, $G_{4}\left(-h_{r}\right)=-\frac{h_{r}}{2}\left[\cos\theta G_{2}^{\prime }\left(-h_{r}\right)+\sin\theta G_{1}^{\prime }\left(-h_{r}\right)\right]-\frac{h_{r}^{2}\sin2\theta }{4}\frac{d^{2}w_{0}^{I}}{dy^{2}}{|}_{y=-h_{r}},$ $G_{5}\left(-h_{r}\right)=-\frac{h_{r}}{2}[\sin\theta G_{2}^{\prime }\left(-h_{r}\right)-\cos\theta G_{1}^{\prime }\left(-h_{r}\right)]+\frac{h_{r}^{2}\cos2\theta }{4}\frac{d^{2}w_{0}^{I}}{dy^{2}}{|}_{y=-h_{r}}$.
